# Comparing sexual self-concept in women with obesity pre- and post-bariatric surgery

**DOI:** 10.1186/s12889-024-19279-y

**Published:** 2024-06-30

**Authors:** Reyhane Ebadifard, Zahra Kiani, Zohreh Keshavarz, Zohre Sheikhan, Mahdi Alemrajabi, Maliheh Nasiri

**Affiliations:** 1 Student Research Committee, School of Nursing and Midwifery, Shahid Beheshti University of Medical Sciences, Tehran, Iran; 2 Midwifery and Reproductive Health Research Center, Shahid Beheshti University of Medical Sciences, Tehran, Iran; 3 Department of Midwifery and Reproductive Health, Midwifery and Reproductive Health Research Center, School of Nursing and Midwifery, Shahid Beheshti University of Medical Sciences, Tehran, Iran; 4 Department of Midwifery, Midwifery and Reproductive Health Research Center, School of Nursing and Midwifery, Shahid Beheshti University of Medical Sciences, Tehran, Iran; 5 Firoozgar hospital, GILDRIC, Iran univetsity of Medical Sciences, Faculty of medicine, Tehran, Iran; 6 Department of Basic Sciences, School of Nursing and Midwifery , Shahid Beheshti University of Medical Sciences, Tehran, Iran

**Keywords:** Sexual self-concept, Obesity, Bariatric surgery

## Abstract

**Background:**

Obesity leads to increased disease burden, decreased life expectancy, and disrupted sexual life. One of the most effective ways of obesity treatment is bariatric surgery. This study was conducted aiming to determine and compare sexual self-concept in women with obesity pre- and post-bariatric surgery.

**Method:**

A longitudinal study comparing sexual self-concept pre and post- surgery was conducted on women with obesity referring to obesity clinics in the city of Tehran in 2020–2021. Data collection was performed using Snell’s Multidimensional Sexual Self-Concept Questionnaire (MSSCQ), which was completed online. Data were analyzed using SPSS version 16 statistical software and Fisher’s exact test, chi-square, Mann-Whitney, independent t, and logistic and linear regression tests. A *p*-value of less than 0.05 was considered significant.

**Results:**

According to the findings, the mean (standard deviation) score of sexual self-concept was 240.26 (26.82) in the post-surgery group and 200.26 (32.24) in the pre-surgery group (*P* = 0.001), and the highest mean (standard deviation) score of sexual self-concept both in the pre-surgery group (13.06 [4.00]) and in the post-surgery group (15.46 [2.16]) was related to the area of sexual depression (*P* = 0.05). Also, with increasing educational level, the odds of bariatric surgery increased by 33%, and those who had no private bedroom had lower odds of bariatric surgery by 65%. In those who did not have other individuals living in their house and their spouse was not a smoker, the self-concept score was 52.35 and 23.11 units higher.

**Conclusion:**

In general, bariatric surgery can improve sexual self-care. Considering the issue of sexual self-concept in bariatric surgery, it is recommended to design appropriate counseling and planning before surgery according to the culture of each country.

## Introduction

Obesity is a crucial global issue that leads to increased disease burden and decreased life expectancy [1]According to the definition of the World Health Organization (WHO), obesity is defined based on a body mass index (BMI) over 30 [2]. In 2016, 39% of adult males and 40% of adult females, almost 2 billion of the world’s adult population, were overweight, and 11% of males and 15% of females all over the world constituted an obese population, whereas both overweight and obesity represent a considerable increase during the last four decades [3]. Obesity leads to the increased possibility of diabetes, high blood pressure, cancer, fatty liver, etc., and even today, it has been recognized that obesity can increase disruptions in sexual desire and sexual [4].

The physical effects of obesity have been studied extensively, but its psychosocial effects have received little attention [5]. Research shows that obesity can influence sexual functioning through increasing anxiety and depression, reducing self-esteem, and debilitating body image [6]. The negative effect of obesity on women’s sexual desires has been well demonstrated [7]. Jiannine stated in a study that 43% of obese women and 31% of obese men suffered from sexual disorders and claimed that sexual self-concept was related to both orgasm and sexual desire [8].

Sexual self-concept is presenting the recognition of an individual’s sexual aspect that culminates in the altered psychological process of individuals in sexual relations and directly affects their sexual functioning [9]. In other words, sexual self-concept means the feelings, ideas, and beliefs of each individual about his/her own sexual relations, which adjusts his/her ideas and beliefs accordingly [10]. On the other hand, body image is considered a crucial aspect of self-concept, and it is significantly related to a positive and negative self-concept so that individuals who are not satisfied with their appearance and physical fitness possess a lower sexual self-concept [11]. Thus, by attenuating body image, obesity disrupts sexual self-concept in obese individuals [8].

There are many ways to treat obesity, one of the most effective of which is bariatric surgery [12]. Bariatric surgery is a type of surgery performed on the structure of the digestive system to treat obesity and includes sleeve gastrectomy (SG), laparoscopic Roux-en-Y gastric bypass (LRYGB), and laparoscopic adjustable gastric banding (LAGB) [13, 14]. Research indicates that the prevalence of sexual dysfunction is higher in women seeking bariatric surgery than other individuals [15].

Różańska-Walędziak et al.’s study showed that women’s sexual functioning post-bariatric surgery had a positive and significant relationship depending on the reduced BMI in all dimensions [16]. In Kolotkin et al.’s study, a positive and significant relationship was also shown between weight loss and improved sexual functioning [7]. However, there are limited studies dealing with the relationship between sexual self-concept and bariatric surgery, and based on the examinations carried out by the researchers of this project, no study was found in this regard in our country. Given the emphasis of the present social standards on women’s fitness, the failure of common obesity treatment methods, and the increasing number of applicants for bariatric surgery, this study was conducted aiming to compare sexual self-concept in two groups of women with obesity pre- and post-bariatric surgery.

## Materials and methods

This longitudinal study was conducted between March 2020 and November 2021 on 242 women with obesity, referring to two obesity clinics in the city of Tehran.

Inclusion criteria included married Iranian 18–50 year-old women with a BMI of 35 and above, having at least reading and writing literacy, being sexually active (having sex at least once a month), having no history of neurological problems (severed spinal cord, paralysis, amputation), psychological problems (depression, personality disorders or depression), and gynecological malignancies (hysterectomy with bilateral oophorectomy, etc.), not consuming narcotics, alcohol, chemical and herbal stimulants, not having spouses with sexual disorders (sexual desire, erectile dysfunction, and ejaculation disorder), and no history of sexual abuse, cardiovascular diseases, chronic diseases such as diabetes and high blood pressure. The participants were also excluded from the study if they did not answer 80% of the questionnaire’s questions.

The sample size in this study was considered 121 people based on the following formula, considering the type I error of 1.96, the type II error of 0.84, the effect size of 0.30, and a significance level of 0.05 and the power of 80%, and taking into account 10% sample loss, 135 people were assigned to each group.

$$\left( {1 - \rho } \right)n \geqslant 2\frac{{{{\left( {{z_{\alpha /2}} + {z_\beta }} \right)}^2}{\sigma ^2}}}{{{{\left( {{\mu _1} - {\mu _2}} \right)}^2}}}$$

$$\alpha = 0.05 \Rightarrow {z_{\alpha /2}} = 1.96$$

$$\beta = 0.20 \Rightarrow {z_\beta } = 0.84$$

$$1 - \beta = 0.80$$

The effect size is the difference between the average score of sexual function and the standard deviation before and after, which was obtained from the article by bond et al. [17].

$${{\left( {{\mu _1} - {\mu _2}} \right)} \mathord{\left/{\vphantom {{\left( {{\mu _1} - {\mu _2}} \right)} \sigma }} \right.\kern-\nulldelimiterspace} \sigma } = 0.30$$

After obtaining permission from the ethics committee of Shahid Beheshti University of Medical Sciences (code: IR.SBMU.PHARMACY.REC.1400.091) and an introduction letter from the university authorities, the researcher went to two obesity clinics in Tehran. A list of women who had six months of surgery and women who were on the surgery list was prepared, and after contacting the patients and checking their specifications, in case of meeting the inclusion criteria, they were explained the study objectives. After obtaining permission (online completion of the informed consent form), the demographic information and midwifery questionnaires and Snell’s Multimedia Sexual Self-Concept Questionnaire (MSSCQ) were provided to the research units in an electronic form (because of the coronavirus disease 2019 (COVID-19) epidemic and traffic restrictions) to be completed within two days.

The participants were two groups of women. The first group included obese women who were candidates for bariatric surgery but had not undergone surgery yet and had received pre-surgery psychiatric counseling, and the second group included women who had at least six months of their bariatric surgery.

The demographic-midwifery questionnaire and Snell’s MSSCQ were used to collect data.

The researcher-made demographic characteristic questionnaire contained 23 demographic questions (age, spouse’s age, duration of the marriage, etc.), 12 reproductive health questions (the number of pregnancies, childbearing, etc.), 12 medical questions (the type of underlying diseases, drugs taken, etc.), and eight questions about habits (cigarette use, hookah use, etc.).

The assessment of the questionnaire’s validity was performed using content validity by presenting the questionnaire to 10 respected faculty members of Midwifery and Reproductive Health Department. The reliability of the questionnaire was measured by the test-retest method (*r* = 0.84).

Snell’s MSSCQ was designed by Professor William Snell in 1998 [18]. This multi-dimensional questionnaire has been designed aiming at self-reporting, which measures 20 psychological aspects of sexual desire by 100 questions, including sexual anxiety sexual self-efficacy, sexual awareness, motivation to avoid high-risk sexual relations, chanciness of sexual behavior control, sexual preoccupation or concern, sexual self-assertiveness, sexual optimism, self-blame in sexual problems, sexual monitoring, sexual motivation, sexual problem management, sexual self-esteem, sexual satisfaction, sexual affair control by others, sexual schema, fear of sexual relations, prevention of sexual problems, sexual depression, and internal sexual control.

Each area contains five questions, which are scored based on a 5-point Likert (Not At All, A Little, Somewhat, Much, and Very Much). Considering the samples’ selected answer, each question is given a score equal to 0–4. Also, a higher score denotes a higher sexual self-concept in each area.

The scale’s Persian version has also been assessed in terms of validity and reliability. In Ramezani et al.’s (2013) [10]study entitled “Assessing the Reliability and Validity of the Multifaceted Sexual Self-Concept Questionnaire in the Iranian Population”, the scientific validity of the questionnaire was approved through content validity, structural validity, and content validity index (CVI), and the reliability of this questionnaire with Cronbach’s alpha coefficient was equal to 0.89 in Ramezani et al.’s (2013) study [10]. The reliability of the questionnaire in the current study was measured by Cronbach’s alpha coefficient equal to 0.94 [10].

After data collection, the data were analyzed using SPSS version 16 statistical software, and Fisher’s exact tests, chi-square, Mann-Whitney, and independent t-tests were performed, and a *p*-value of less than 0.05 was considered significant. Also, to assess the relationship of demographic factors with bariatric surgery and sexual self-concept, logistic and linear regression were used so that univariate analysis was initially performed, and then significant variables (*P* < 0.1) were entered into the model, and linear regression was carried out.

## Results

Two hundred and forty-two married women meeting the inclusion criteria participated in this research and completed all the relevant questionnaires. First, there were 135 people in each group, which, unfortunately, 8 people from the pre-surgery group and 10 people from the post-surgery group were excluded due to not completing 80% of the questionnaire’s questions, 6 people from the pre-surgery group withdrew from the research because of the cancellation of their surgery due to COVID-19, and 4 people from the pre-surgery group withdrew from the research at the time of statistical analysis; so, a total of 117 people from the pre-surgery group and 125 people from the post-surgery group were included in the study.

Average age and duration of marriage of the pre and post-surgery 37.29 ± 7.15, 38.17 ± 6.83 and15.06 ± 8.78, 16.00 ± 8.42, respectively. In the current study, most of the participants were. The mean BMIs of the participants were 41.82 ± 5.99 in the pre-surgery group and 27.00 ± 4.56 in the post-surgery group (Table 1).

**Table 1:** Demographic characteristics of the participants

Variable Group	Pre-Surgery Group		Post-Surgery Group		*P*-Value
	Mean	Standard Deviation	Mean	Standard Deviation	
Age	37.29	7.15	38.17	6.83	0.33*
Spouse’s age	41.35	8.06	42.85	8.66	0.16*
Body mass index	41.82	5.99	27.00	4.56	0.00*
Age at the time of marriage	22.23	5.31	22.04	4.18	0.75*
Duration of marriage	15.06	8.78	16.00	8.42	0.39*
Number of sexual relations per month	5.76	4.26	5.60	3.98	0.76**

* Independent t

** Mann-Whitney

The levels of family income adequacy in the two groups had a statistically significant difference and were higher in the post-surgery group (*p* = 0.000) (Table 2).

**Table 2:** Demographic characteristics of the participants

Groups Variable		Pre-Surgery Group	Post-Surgery Group	*P*-Value
		Number (Percentage)	Number (Percentage)	
Type of marriage	Imposed	1 (0.09)	2 (1.6)	0.23*
	Traditional	53 (45.3)	69 (55.2)	
	Previous acquaintance	63 (53.8)	54 (43.2)	
Ethnicity	Fars	79 (82.9)	113 (90.4)	0.85**
	Not Fars	20 (17.1)	12 (9.6)	
Education	Under diploma	21 [18]	4 [5]	0.01***
	Diploma	38 (32.5)	42 (33.6)	
	Associate	9 (7.7)	13 (10.4)	
	Bachelor	38 (32.5)	40 (32)	
	Master and higher	11 (9.4)	25 [19]	
Job	Working at home	12 (10.3)	8 (6.4)	0.58*
	Working outside the home	38 (32.5)	43 (34.4)	
	Housewife	66 (56.4)	71 (56.8)	
	Student	1 (0.9)	3 (2.4)	
Spouse’s job	Unemployed	1 (0.9)	0 (0)	0.53*
	Employee	41 (35)	52 (41.6)	
	Student	1 (0.9)	0 (0)	
	Self-employed	74 (63.2)	73 (58.4)	
Income adequacy	Adequate	82 (70.1)	105 (84)	0.000**
	Lower than adequacy	27 (23.1)	7 (5.6)	
	Higher than adequacy	8 (6.8)	13 (4.10)	
Other individuals living in the house	Yes	11 (9.4)	11 (8.8)	0.87**
	No	106 (90.6)	114 (91.2)	
Private bedroom	Has	98 (83.8)	119 (95.2)	0.003**
	Does not have	19 (6.2)	6 (4.8)	
Narcotic use in spouse	Has	2 (1.7)	3 (2.4)	0.70*
	Does not have	115 (98.3)	122 (97.6)	
Cigarette use	Has	2 (1.7)	1 (0.8)	0.52*
	Does not have	115 (98.3)	124 (99.2)	
Cigarette in spouse	Has	44 (37.6)	44 (35.2)	0.69**
	Does not have	73 (62.4)	81 (64.8)	

* Fisher’s exact test

** Chi-square test

*** Mann-Whitney test

The result of the independent t-test between the mean scores of sexual self-concept in the two groups pre- and post-bariatric surgery in the areas of sexual anxiety, sexual self-efficacy, sexual awareness, chanciness of sexual behavior control, sexual preoccupation or concern, sexual optimism, self-blame in sexual problems, sexual motivation, sexual management, sexual satisfaction, sexual schema, fear of sexual relations, and sexual affair control were higher in the post-surgery group than in the pre-surgery group (*P* < 0.05), and no statistically significant difference was observed between the two groups in other areas (Table 3).

**Table 3:** Comparing the sexual self-concept variable and its dimensions in two groups pre- and post-bariatric surgery

Variable	Mean (Standard Deviation) Pre-Surgery (*N* = 117)	Mean (Standard Deviation) Post-Surgery (*N* = 125)	*P*-Value
Sexual anxiety	10.26 (1.43)	11.53 (1.55)	0.02
Sexual self-efficacy	7.00 (2.97)	10.33 (3.03)	0.005
Sexual awareness	12.13 (3.04)	14.66 (1.83)	0.01
Motivation to avoid high-risk sexual relations	12.20 (0.77)	13.13 (2.35)	0.15
Chanciness of sexual behavior control	10.93 (2.34)	12.66 (1.49)	0.02
Sexual preoccupation or concern	8.00 (1.64)	9.66 (1.67)	0.01
Sexual self-assertiveness	12.46 (3.02)	14.20 (2.27)	0.08
Sexual optimism	9.20 (2.00)	10.86 (1.76)	0.02
Self-blame in sexual problems	8.46 (2.66)	10.60 (2.41)	0.02
Sexual monitoring	9.13 (1.76)	10.66 (2.52)	0.06
Sexual motivation	11.33 (1.79)	13.40 (2.09)	0.007
Sexual problem management	9.53 (3.20)	12.66 (2.58)	0.006
Sexual self-esteem	10.66 (2.09)	12.00 (1.06)	0.03
Sexual satisfaction	5.66 (2.35)	7.86 (2.82)	0.02
Sexual Schemas	8.93 (3.45)	12.26 (2.49)	0.005
Fear of sexual relations	12.20 (2.88)	14.46 (2.50)	0.02
Sexual affair control by others	9.13 (2.66)	12.13 (2.92)	0.007
Prevention of sexual problems	10.26 (2.31)	11.20 (1.82)	0.23
Sexual depression	13.06 (4.00)	15.46 (2.16)	0.05
Sexual internal control	9.66 (2.82)	10.46 (2.29)	0.40
Total	200.26 (32.24)	240.26 (26.82)	0.001

The present study also indicated that by increasing the educational level, the odds of bariatric surgery increased by 33%, and those who had no private bedroom had lower odds of surgery by 65% (Table 4).

**Table 4:** Assessing the relationship between bariatric surgery and demographic factors

	Estimation Amount	Standard Deviation	Wald Statistic	Degree of Freedom	*P*-Value	Odds Ratio	Upper Bound	Lower Bound
Type of marriage	-0.568	0.262	4.692	1	0.030	0.567	0.339	0.947
Ethnicity	-0.655	0.405	2.614	1	0.106	0.520	0.235	1.149
Education	0.284	0.097	8.626	1	0.003	1.328	1.099	1.605
Private bedroom	-1.062	0.513	4.281	1	0.039	0.346	0.126	0.946

Linear analysis

Furthermore, the self-concept score of those who had other individuals living in their house or who did not have smoker spouses was 52.35 and 23.11, scores higher (Table 5).

**Table 5:** Assessing the relationship between the total score of sexual self-concept and demographic factors

	Estimation Amount	Standard Deviation	Beta Standardized Estimation	t Statistic	*P*-Value
Group	20.718	10.140	0.296	2.043	0.052
Other individuals living in the house	52.346	19.373	0.374	2.702	0.012
Spouse’s cigarette use	23.112	10.687	0.319	2.163	0.040
Job	-5.690	9.591	-0.080	-0.593	0.558

Lineal analysis

## Discussion

The present study showed that the total score of sexual self-concept and some of its areas, such as sexual anxiety, sexual self-efficacy, sexual awareness, chanciness of sexual behavior control, sexual preoccupation or concern, sexual optimism, self-blame in sexual problems, sexual motivation, sexual management, sexual satisfaction, sexual schemas, fear of sexual relations, and sexual affair control were higher in the post-surgery group, and the highest and lowest difference in the mean score of sexual self-concept in both the pre-surgery and post-surgery groups was related to sexual depression and sexual satisfaction, respectively. In Rojas (2011) and Morais’s (2011) studies, the total mean score of self-concept had reduced 6 months and 3 months post-bariatric surgery, respectively, but these changes were not statistically significant [19, 20], whereas the mean score of sexual self-concept in the current research reached from 200.26 pre-surgery to 240.26 during 6 months post-bariatric surgery, and was also statistically significantThis difference is due to the different sample size and research location. Overall, based on the results of various studies and since promoting sexual self-concept is affected by numerous factors in individuals’ lives, including biological (weight loss, physical attractiveness, etc.), psychological (mental image, self-confidence, etc.), and social factors (family and occupational roles), the integrated implementation of health policies is essential to improve sexual self-concept [21–24].

Steinke et al.’s (2008) study concluded that [25], a higher sexual self-concept from greater sexual self-efficacy and lower sexual anxiety, younger age, and being married were significant predictors of sexual activity. Jolfaei et al.’s (2016) study indicated that Sexual performance improved and self-esteem did not find a significant difference [26].

In the current research, the mean scores of the areas of sexual problem management, sexual problem monitoring, and sexual affair control by others in the post-surgery women group were in the unfavorable range, but in the group of women after bariatric surgery, all these areas were in the favorable range. Similarly, the different mean scores of these areas between the two groups of pre-surgery and post-surgery were statistically significant. The results of the present research are in line with the results of Azizi’s (2020) study [27].

The current study indicated that by increasing the educational level, the odds of bariatric surgery increased by 33%, those who had no private bedroom had lower odds of surgery by 65%, and those who did not have other individuals living in their house or whose spouse was not a smoker, had higher sexual self-concept scores.

Çaynak et al.’s study, investigating the effects of body perception and sexual desires of bariatric surgery patients on obese individuals referred for bariatric surgery, indicated that BMI, height, and weight were not significantly related to sexual self-concept [28]. The result of this study is consistent with the current study. Ternas’s study (2020) in Iran has shown the significant relation of women’s BMI to their sexual self-concept and sexual functioning [29]. The results of this study are not in line with the present study. The reason for the inconsistent results of these two studies may be due to different target populations, so that in the present study, the participants’ mean age was 37 years, while in the above study, more than 61% of the participants aged less than 25 years. The higher age and experience of the sexual lives of the participants of the present study may help them to be more sexually compatible.

In Lotfollahi et al.’s study comparing sexual self-concept in fertile and infertile women, it was observed that sexual satisfaction and fear of sexual relations were significantly lower in infertile women than in fertile women, indicating the effect of fertility on sexual self-concept [30]. This study is also inconsistent with the present study. The reason for the inconsistency between the results of the above study and that of the present study may be that in the present study, more than 85% of the participants had experienced pregnancy and childbirth.

Martin et al.’s (2010) study demonstrated that socioeconomic factors played a critical role in performing bariatric surgery in medically qualified individuals so that the likelihood of surgery was higher in individuals with higher educational levels and higher income than others [31]. The results of this study support the results of the present study.

Considering the limited number of studies on the relationship between sexual self-concept and demographic factors, more studies are required.

Among the strengths of this research is its novelty and lack of similarity with other studies in terms of the use of tools and variables that impact women’s sexual self-concept. Comparing the two groups of women pre-surgery and post-surgery, this research helped better understand sexual problems in obese women and the effect of surgery on their problems.

Among the limitations of this project were cultural reasons and the topic sensitivity, which made it possible for the subjects under investigation to avoid expressing sexual problems or unusual types of sexual relations. The COVID-19 outbreak and the stress and anxiety in the subjects also led to negative effects on the research results.

Since the difference in the mean score of sexual self-concept between the two groups of women pre-and post-bariatric surgery was significant, it is suggested that the effect of bariatric surgery be assessed on a wider sample with the participation of women and men in other cities to generalize it to the whole country. Since, despite the researcher’s search, no interventional or descriptive study was unfortunately found to investigate the level of sexual self-concept in individuals with a history of bariatric surgery, and the effect of bariatric surgery on sexual self-concept has not been recognized yet, it is suggested that such a study be performed with the participation of individuals from both male and female genders, and the effect of bariatric surgery on sexual self-concept be also compared with other weight loss treatments.

### Limitation

One of the main limitations of this study was the sampling during the COVID-19 and the reduction of visits to the hospital for surgery, and this caused the sample before and after surgery to be different. We tried to solve this problem by matching of groups and statistical methods.

### Strength

It is one of the first studies in Iran in this field, with a suitable and generalizable sample size. This study can become the basis of hypotheses and intervention planning.

## Conclusion

Sexual self-concept in obese people is related to various factors. According to the results of the current research, bariatric surgery can improve sexual self-care. Considering the issue of sexual self-concept in bariatric surgery, it is recommended to design appropriate counseling and planning before surgery according to the culture of each country.

## Data Availability

The data that support the findings of this study are available from Zohre keshavarz but restrictions apply to the availability of these data, which were used under license for the current study, and so are not publicly available.

## Abbreviations

BMI: Body Mass Index
SG: Sleeve gastrectomy
LRYGB: laparoscopic Roux-en-Y gastric bypass
LAGB: laparoscopic adjustable gastric banding
COVID-19: Coronavirus Disease 2019
WHO: World Health Organization
SPSS: Statistical Package for the Social Sciences
SD: Standard Deviation
